# Wearable and Portable Devices in Sport Biomechanics and Training Science

**DOI:** 10.3390/s24144616

**Published:** 2024-07-17

**Authors:** Diego Jaén-Carrillo, Alejandro Pérez-Castilla, Felipe García-Pinillos

**Affiliations:** 1 Department of Sport Science, University of Innsbruck, 6020 Innsbruck, Austria; 2 Department of Education, Faculty of Education Sciences, University of Almería, 04120 Almería, Spain; alexperez@ual.es; 3 SPORT Research Group (CTS-1024), CIBIS (Centro de Investigación para el Bienestar y la Inclusión Social) Research Center, University of Almería, 04120 Almería, Spain; 4 Department of Physical Education and Sport, University of Granada, Carretera de Alfacar 21, 18011 Granada, Spain; fgpinillos@ugr.es; 5 Sport and Health University Research Center (iMUDS), Parque Tecnologico de la Salud, Av. Del Conocimiento s/n, 18007 Granada, Spain; 6 Department of Physical Education, Sport and Recreation, Universidad de La Frontera, Temuco 01145, Chile

Sport biomechanics and training have traditionally been tested under laboratory conditions, requiring specific settings and expensive equipment [1,2]. The novel use of wearable devices addresses the lack of ecological validity in such measures and offers an affordable, user-friendly option for biomechanical assessments [3,4,5]. Recently, wearable sensors have enabled the quantification of performance and workload by providing mechanical and physiological parameters, leading to their exponential growth in popularity [3,5]. Many wearable sensors are now commercially available and capable of delivering both kinetic and kinematic data, thus improving the feasibility and efficiency of assessments and making them a viable alternative for sports practitioners and researchers [5]. Additionally, wearable devices allow for real-time monitoring and biofeedback [6]. This Special Issue of *Sensors* aims to provide current information about the use and application of wearable sensors in sport biomechanics and training science.

This Special Issue features twelve original articles (contributions 1–12) and a systematic review (contribution 13).

Contribution Summaries

*Biomechanical Changes in Running Shoes*: A study (contribution 1) investigated biomechanical changes in the lower limbs when transitioning from level ground to an uphill slope with different levels of longitudinal bending stiffness (LBS) in running shoes. The authors concluded that running uphill with high LBS shoes improves lower limb efficiency but increases knee joint energy absorption, potentially raising the risk of knee injuries. Amateurs should choose running shoes with optimal stiffness. 

*Metabolic Power and Energy Cost in Sprinting*: Contribution 2 compared methods for calculating the metabolic power (MP) and energy cost (EC) of sprinting using GPS metrics and electromyography (EMG). The study found that GPS-based calculations underestimated neuromuscular and metabolic engagement, suggesting that EMG-derived methods are more accurate for MP and EC calculations during sprints.

*Assessment of Torque–Velocity Profile in Cycling*: One study (contribution 3) aimed to determine the feasibility, test–retest reliability, and long-term stability of a novel method for assessing the torque–velocity profile and maximal dynamic force (MDF) during leg-pedaling. Using a friction-loaded isoinertial cycle ergometer and a high-precision power meter, the data supported the method’s validity, reliability, and long-term stability for cyclists.

*Motor Development and Training*: This empirical study (contribution 4) tested the assumed hierarchy for learning skills in motor development and training literature. The authors concluded that the proposed learning hierarchy is valid for some tasks, although the underlying reasons remain unknown.

*Reliability of Wearable Systems*: Four contributions (5, 10, 11, and 12) examined the reliability and agreement levels of different wearable systems:

- Contribution 5 evaluated the reliability of Xsens-based lower extremity joint angles during running on stable and unstable surfaces. The system captured within-day kinematic adaptations but showed less reliability for between-day measurements, particularly for ankle and hip joint angles in the frontal plane.
- Contribution 10 compared a markerless motion capture system (MotionMetrix) with an optoelectronic MCS (Qualisys) for kinematic evaluations during walking and running. The agreement varied by variable and speed, with some variables showing high agreement and others poor agreement.
- Contribution 11 assessed the validity of the three-sensor RunScribe Sacral Gait Lab™ IMU for measuring pelvic kinematics compared to the Qualisys system. The IMU did not meet the validity criteria for any variables or velocities tested.
- Contribution 12 analyzed the test–retest and between-device reliability of the Vmaxpro IMU for estimating vertical jump. The Vmaxpro was deemed unreliable for measuring vertical jumps.

*Effects of Pressurization Modes on Muscle Activation*: Contribution 6 examined the effects of different pressurization modes during high-load bench press training on muscle activation and subjective fatigue in bodybuilders. High-intensity bench press training with either continuous or intermittent pressurization significantly increased muscle activation, with continuous pressurization resulting in higher perceived fatigue.

*Real-Time Monitoring in Fencing*: Contribution 7 assessed the efficacy of a novel system for real-time monitoring of fencers’ balance and movement control, enhanced by visual and haptic feedback modules. The findings suggest that integrating the Internet of Things (IoT) and real-time sensory feedback improves performance in fencing.

*Thermoregulation in Athletes*: A study (contribution 8) described bilateral variations in skin temperature of the anterior thigh and patellar tendon in healthy athletes, providing a model of baseline thermoregulation following a unilateral isokinetic fatigue protocol. The thermal challenge produced homogeneous changes in the quadriceps but not in the tendon areas, indicating that metabolic and blood flow changes depend on the tissue’s physical and mechanical properties.

*Discriminatory Power of Physical Tests*: Contribution 9 examined whether specific physical tests can differentiate players with similar anthropometric characteristics but different playing levels. The authors concluded that a combination of the specific performance test and the force development standing test effectively identifies talent and differentiates between elite and sub-elite players.

*Systematic Review on Beach Invasion Sports*: The systematic review (contribution 13) aimed to (1) characterize internal and external loads during beach invasion sports, (2) identify monitoring technologies and metrics, (3) compare demands with indoor sports, and (4) explore differences by competition level, age, sex, and beach sport. Key findings demonstrated that beach sports involve moderate-to-high-intensity bouts with lower-intensity recovery, the unstable sand surface and variable outdoor conditions increase perceptual effort despite lower external load volumes compared to indoor sports, and substantial variability exists in acceleration, impact, and internal load intensity zones in the limited beach sports research.

Conclusions

In summary, this Special Issue provides valuable insights into sports biomechanics and training science, particularly the application of wearable devices for real-time monitoring and biofeedback. 
